# 3D printing in palliative medicine: systematic review

**DOI:** 10.1136/bmjspcare-2021-003196

**Published:** 2022-09-06

**Authors:** Tjaša Kermavnar, Callum Guttridge, Niall J Mulcahy, Ed Duffy, Feargal Twomey, Leonard O'Sullivan

**Affiliations:** 1 Health Research Institute, School of Design, and Confirm Smart Manufacturing Centre, University of Limerick, Limerick, Ireland; 2 Deparment of Palliative Medicine, Milford Care Centre Castletroy, Limerick, Ireland; 3 Palliative Medicine, Milford Hospice, Limerick, Ireland

**Keywords:** chronic conditions, hospice care, hospital care, quality of life

## Abstract

**Background:**

Three-dimensional printing (3DP) enables the production of highly customised, cost-efficient devices in a relatively short time, which can be particularly valuable to clinicians treating patients with palliative care intent who are in need of timely and effective solutions in the management of their patients’ specific needs, including the relief of distressing symptoms.

**Method:**

Four online databases were searched for articles published by December 2020 that described studies using 3DP in palliative care. The fields of application, and the relevant clinical and technological data were extracted and analysed.

**Results:**

Thirty studies were reviewed, describing 36 medical devices, including anatomical models, endoluminal stents, navigation guides, obturators, epitheses, endoprostheses and others. Two-thirds of the studies were published after the year 2017. The main reason for using 3DP was the difficulty of producing customised devices with traditional methods. Eleven papers described proof-of-concept studies that did not involve human testing. For those devices that were tested on patients, favourable clinical outcomes were reported in general, and treatment with the use of 3DP was deemed superior to conventional clinical approaches. The most commonly employed 3DP technologies were fused filament fabrication with acrylonitrile butadiene styrene and stereolithography or material jetting with various types of photopolymer resin.

**Conclusion:**

Recently, there has been a considerable increase in the application of 3DP to produce medical devices and bespoke solutions in the delivery of treatments with palliative care intent. 3DP was found successful in overcoming difficulties with conventional approaches and in treating medical conditions requiring highly customised solutions.

**Key messages**

**What was already known?**

- Specialists in palliative medicine often require short term, rapid solutions to alleviate the patients’ distressing symptoms and improve their quality of life. Three-dimensional printing (3DP) is becoming more common to manufacture complex patient-specific devices and is recognised for its ability to provide cost-effective and customisable rapid solutions. Patients in receipt of palliative care can benefit from the advantages of 3DP; but in order to highlight potential opportunities, it is necessary to systematically review its use in this clinical field.

**What are the new findings?**

- The majority of reports of 3DP use in palliative care were published after the year 2017. The studies showcase a versatile range of potential applications, including for the production of anatomical models, endoluminal stents, navigation guides, obturators, epitheses, endoprostheses and others. The main reasons for using 3DP are the difficulty of producing patient-specific devices with traditional methods, and the lack of commercially available solutions to specific patient needs.

**Key messages**

**What is their significance?**

Clinical

- Using 3DP-generated applications as a component of the care provided to patients with palliative care needs can lead to a positive impact on palliative care patient outcomes, particularly when cost, time and the possibility of customisation are critical factors. Guidelines are provided regarding the advantages and disadvantages of specific 3DP technologies and materials, both to inform future clinical practice and identify limitations.

Research

- To the authors’ knowledge, this study is the first comprehensive systematic review analysing 3DP as a method of producing medical devices that might be applied to patients receiving palliative care.

## Introduction

 Three-dimensional printing (3DP), also known as additive manufacturing (AM), is becoming increasingly common in modern medicine. Initially, it was limited to manufacturing prototypes, and was synonymous with rapid prototyping (RP),^1^ but it is being increasingly used to directly produce finished products and components.^2^ Physical objects are built from digital data (ie, computer-aided design models) that can be generated anew using 3D-modelling software, or obtained by 3D-scanning of existing objects in the process of reverse engineering (RE). The final designs are then 3D-printed directly (direct AM), or fabricated with the help of 3D-printed tools/moulds (indirect AM).

Presently, 3DP is gaining increasing recognition in a range of medical practices, including diagnostics, surgical planning and reconstruction, patient education, rehabilitation, tissue engineering and pharmacology.^3^ In the production of medical devices and tools, 3DP offers a wide range of advantages over traditional methods, most notably the possibility of cost-effective, small-scale, on-demand, in-house fabrication of geometrically and structurally complex patient-specific products in a relatively short time.^4 5^ These advantages can add particular value to the delivery of responsive care to patients with palliative care needs. Namely, the possibility of producing highly customised solutions at low cost allows for individualised management of patients’ needs to help them cope with their condition and treatment, and experience optimal quality of life despite the disease. Moreover, reduced lead time enables a quick response to alleviating distressing symptoms and allow a person whose health is deteriorating to spend less time away from their home.

It is of note, that in part due to the relatively recent recognition of palliative medicine as a specialty, even among healthcare professionals a common understanding of the roles of palliative care still needs to be established.^6 7^ To facilitate this, the International Association for Hospice and Palliative Care published a new ‘Consensus-Based Definition of Palliative Care’ in 2019.^8^ For clarity, the authors of the present work also acknowledge the following: (1) specialist palliative care is given alongside treatments targeting the underlying disease; (2) when the intention is potentially curative, the intervention does not qualify as truly palliative and (3) interventions provided with palliative intent are typically less invasive and less dangerous procedures, although the same medical approaches can have curative effects in some diseases, and palliative in others (eg, central airway obstruction management with stents,^9^ radioactive ^125^I seed implantation for brachytherapy,^10^ bone tumour resection^11^ and endoprosthetic reconstruction^12^).

Individual literature reviews exist of 3DP in palliative care, focused on specific types of medical devices, such as central airway stents,^9^ oesophageal stents^13^ and orthoses.^14^ However, to the authors’ knowledge, no systematic reviews have been published to date in this field. Thus, the aim of this study is to provide a systematic review of studies reporting the use and the potential uses of 3DP in specialist palliative care, with specific emphasis on the fields of application, technology employed and the advantages of 3DP over conventional methods.

## Methods

### Literature search and study selection

A systematic literature search was performed during December 2020 using the following databases: EBSCOhost (including Academic Search Complete, MEDLINE with Full Text, CINAHL Complete), PubMed, Scopus, and Web of Science. Articles of interest included terms related to 3DP in the title (ie, “3D print*“,“3D-print*“, “three-dimensional* print*“, “additive* manufactur*“, or “rapid* prototyp*“), terms related to palliative care in the abstract (ie, “palliat*“, “cancer*“, “oncolog*“, “tumour*“, “tumor*“, “malignan*“, “terminal* ill*“, or “terminal* disease*“), and terms related to palliative care in the full text (ie, “palliat* car*“, “palliat*“, “end-of-life”, “end of life”, “quality-of-life”, or “quality of life”). If necessary, the search string was adapted to meet the search options of specific databases. An additional search was performed using Scopus to identify studies including any of the terms related to 3DP and the term “palliative” in either the title, abstract or keywords. The study selection was limited to full scientific articles in the English language. All included papers were published prior to the date of the search. Reviews, book chapters and non-scientific papers were excluded from the review, as were studies performed on veterinary patients, involving curative or aesthetic surgical reconstructive procedures, and testing diagnostic technology. Also excluded were studies involving palliative surgical correction of paediatric congenital heart defects, as these are typically managed by cardiologists. Regarding bias, all studies which met the selection criteria were included.

**Figure 1:**
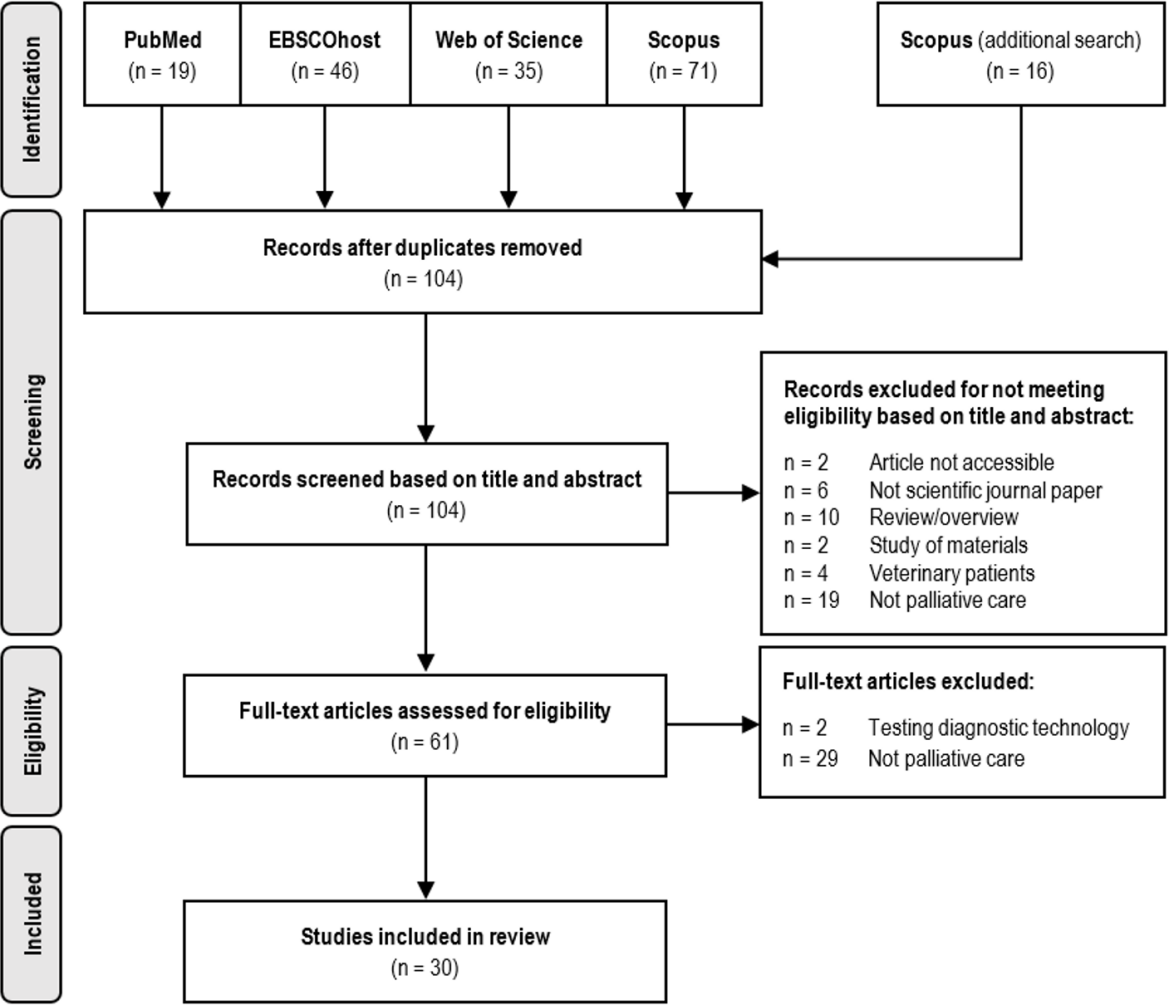
PRISMA flow diagram of literature search and study selection. PRISMA, Preferred Reporting Items for Systematic Reviews and Meta-Analyses.

The review protocol was designed according to the Preferred Reporting Items for Systematic Reviews and Meta-Analyses guidelines.^15^ The search and study selection criteria are presented in figure 1. TK confirmed the outcomes of the search and selection performed by NJM and CG. Any disagreements among the reviewers were resolved by LOS.

### Data extraction and synthesis

The following data were extracted from the selected studies: (1) field of application of 3DP in palliative care, type of 3D-printed device, its stage of development and application; (2) technology used for device fabrication including 3DP technology, 3D-printer make, material, imaging technique, software used and (3) testing of the 3D-printed device, including number of participants, age and medical status, testing method and outcomes of intervention. 3D-printed device manufacturer, print time and cost were also reviewed.

## Results

**Figure 2:**
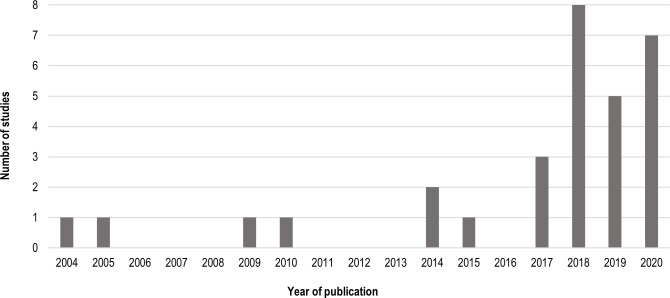
Reviewed studies involving the use of 3DP in palliative care by year of publication. 3DP, three-dimensional printing.

Thirty relevant papers on the use of 3DP in palliative care were identified and included in the review. The first study was published in 2004, and 20 papers were published in the last 3 years, as shown in figure 2.

### Device type and field of application

**Figure 3:**
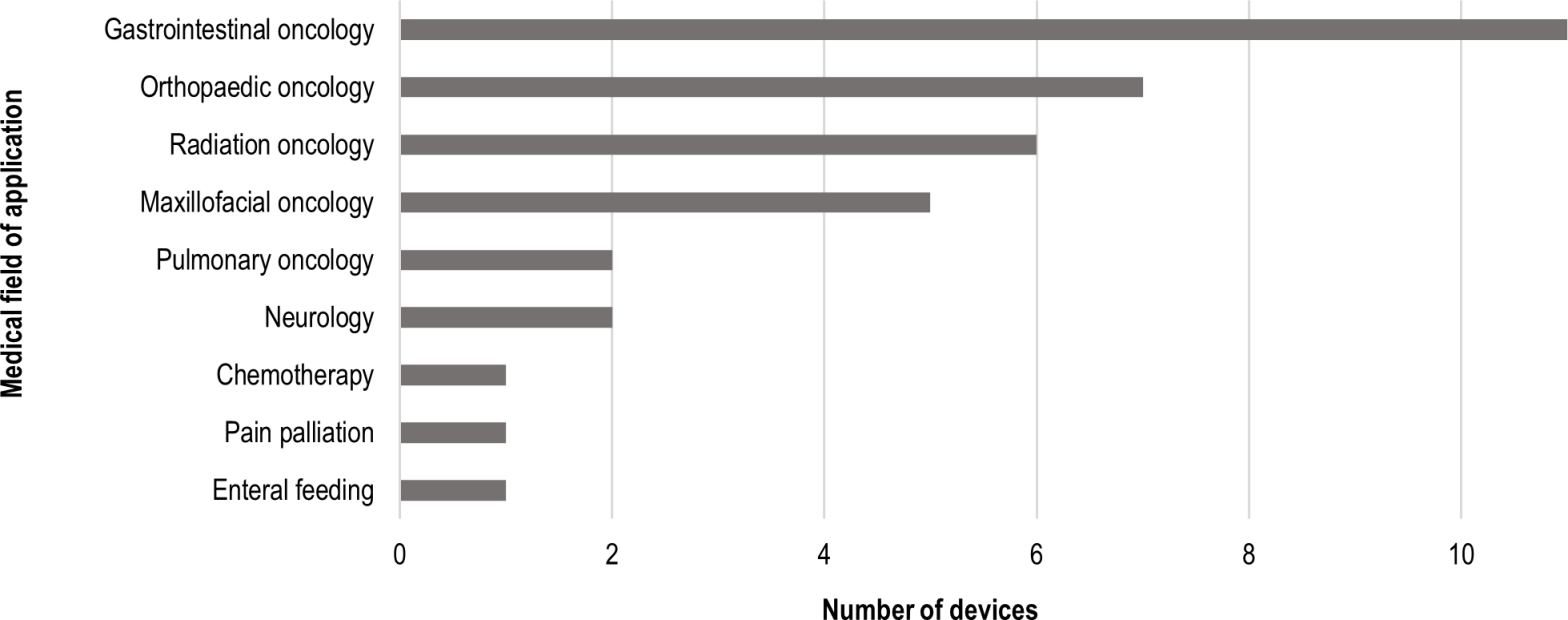
Fields of application of 3DP in palliative care. 3DP, three-dimensional printing.

3DP was applied to different medical sub-specialties within oncology, predominantly gastrointestinal, orthopaedic and radiation oncology. Only three devices were produced for non-oncological applications (figure 3).

**Figure 4:**
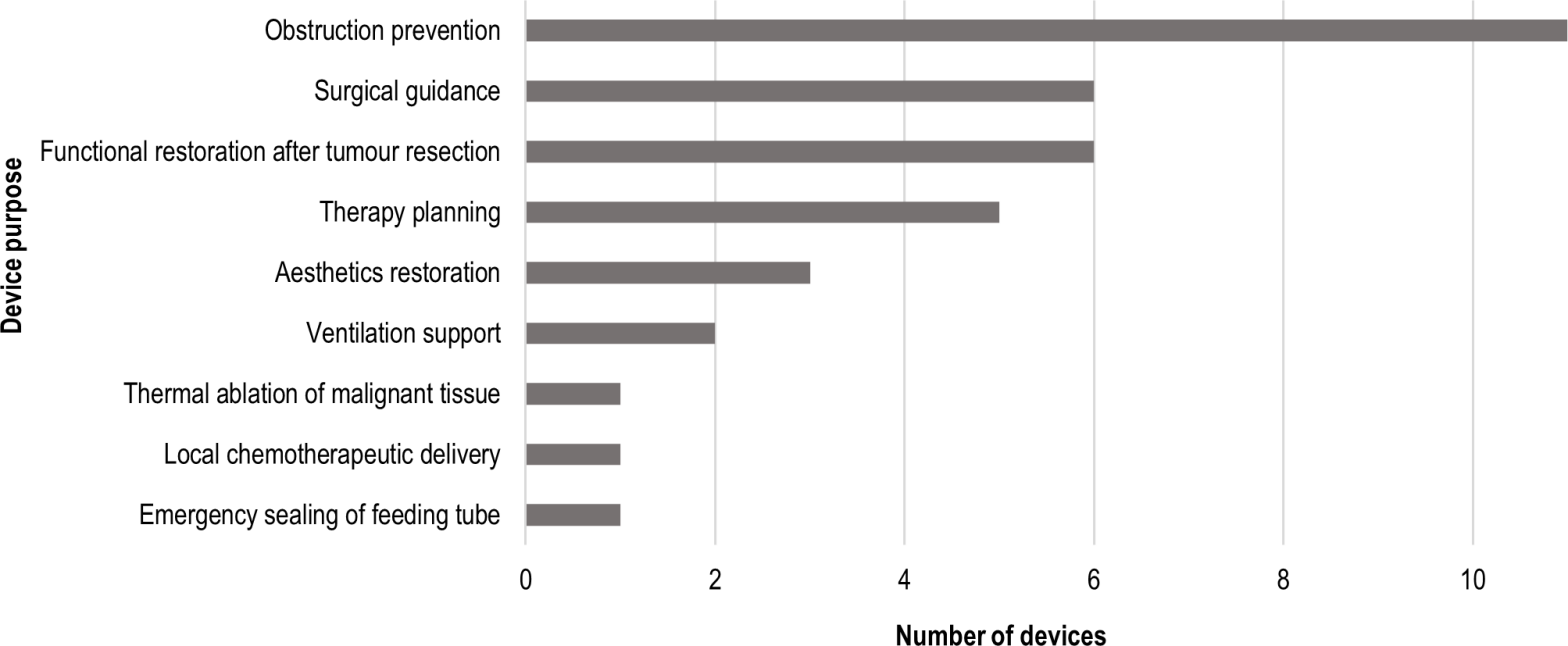
Purpose of the reviewed 3D-printed devices in palliative care. 3D, three dimensional.

In the 30 reviewed studies, 36 different devices were produced (online supplemental table l). The most common were endoluminal stents (9), however, all were used in proof-of-concept studies. Other most commonly 3D-printed devices were anatomical models (6), brachytherapy navigation guides (5), endoprostheses (including one mould; 4), epithesis casts and moulds (3) and obturator casts (2). In single cases, an injection-moulding chamber, surgical cutting guide, PEG-tube sealing device, respirator mask and positive mould, scaffold for chemotherapeutic delivery, and a robot for ultrasound pain palliation were manufactured. Figure 4 summarises the purpose of the devices.

### Problems addressed by 3DP

**Figure 5:**
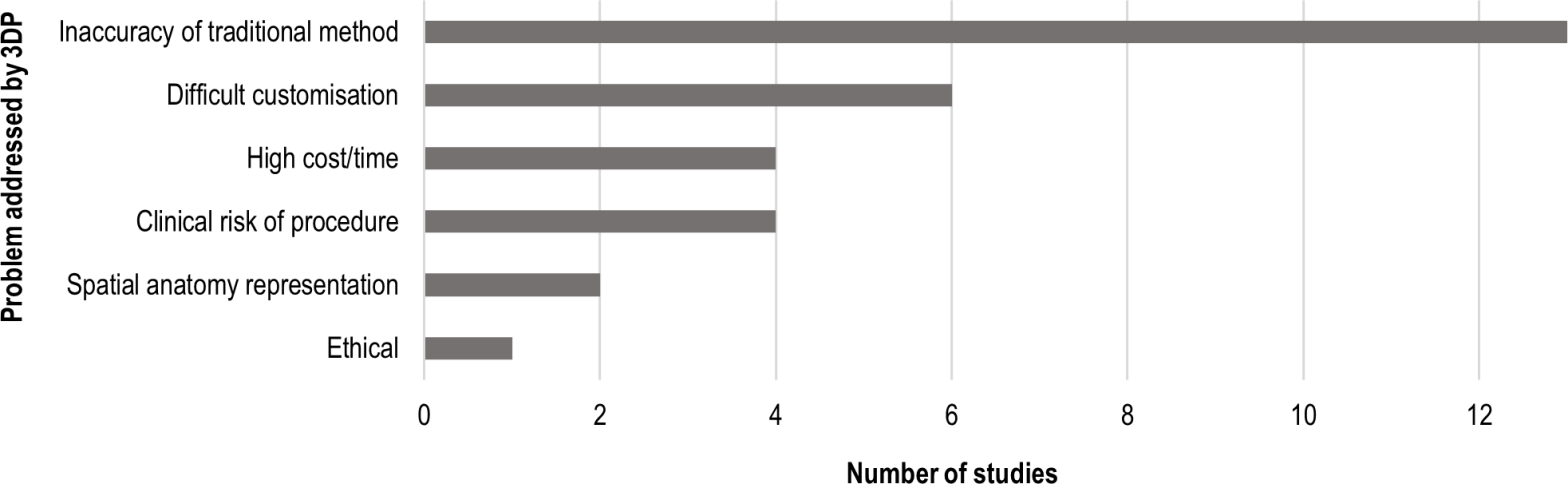
Problems addressed by 3DP in the reviewed studies. 3DP, Three-Dimensional Printing.

The most common purpose of 3DP was to improve the accuracy and/or efficiency of treatment achievable with traditional methods (13 devices). Seven of the 13 devices were intended to improve the accuracy of drug delivery, 2 were endoluminal stents with improved patency or drug distribution, and 1 was an anatomical model for improved surgical planning. A further three devices were used to address the lack of efficiency in the traditional method (ie, one cutting guide, one endoprosthesis, one obturator mould). In six studies, 3DP was chosen to address the difficulty of device customisation with traditional methods, including endoluminal stents (3), endoprosthesis (1), epithesis (1) and respirator mask (1). In two studies, 3D-printed anatomical models were used to address difficulties of spatial anatomy comprehension from 2D images. Four devices were used to reduce the risks for patients associated with conventional methods, and one anatomical model was used as an alternative to human testing (figure 5).

3DP was used with the intention to reduce the cost and manufacturing time of two epitheses and two endoluminal stents. Time-efficiency was reported in four studies with print durations ranging up to to 36 hours,^16^ and two studies highlighted the potential for delivering custom 3D-printed devices to patients within 24 hours.^17 18^ Cost-effectiveness of 3DP was emphasised in two cases (US$30 for a head mould to replace a US$200–US$400 CT scan^16^ and a US$5 custom-fit BiPAP mask^16^), and one study considered the price disadvantageous (US$500 for materials and printing of an obturator definitive cast).^19^

### 3DP technology

Thirteen devices were manufactured using Fused Filament Fabrication (FFF),^1620^,^30^ one of which used a custom built FFF gantry specifically designed for the orbital printing of stents.^26^ Six devices were produced using StereoLithogrAphy (SLA),^1822 31^,^33^ five using Material Jetting (MJ),^1719 34^,^36^ two using Selective Laser Sintering (SLS)^18 33^ and in single cases, direct metal laser melting^37^ and electron beam melting^33^ were employed. One study reported the use of selective laser lithography^12^ (the authors of the present review are unfamiliar with this technology). In seven studies, 3DP technology was not specified; however, four of these detailed the type of material used (ie, photopolymer resin, medical resin, and PolyMethyl MethAcrylate (PMMA)).

Ten of the reviewed papers did not detail the material employed. Across the other studies, the most common materials were photopolymer resin (including Flexible Resin, MED610, Tango family and VisiJet C4 Spectrum Core; 8) used with MJ or SLA, and Acrylonitrile Butadiene Styrene (ABS; 6) used with FFF. Also employed were Polycaprolactone (PCL, including in combination with Paclitaxel—PCL/PTX; 2), PolyLActic acid (PLA, including in combination with thermoplastic polyurethane—PLA/TPU; 2), polymethyl methacrylate (PMMA; 2), PolyVinyl Alcohol (PVA, including in combination with TPU—TPU/PVA; 2), Titanium alloy (2) and Polyurethane (PU; 1).

Patient-specific devices were mainly reverse engineered, which involved surface 3D scanning or CT/MRI, and designing the device based on the digital data of patients' anatomy. Devices that were directly designed included nine endoluminal stents, not tested on patients, a coplanar navigation guide, PEG tube sealing device, scaffold for chemotherapeutic delivery and robotic system for ultrasound palliation of pain. Unlike RE, these devices were designed independently of the specific patients’ anatomy. Indirect AM was used to create moulds for obturators, epitheses and respirator masks manufactured from silicone; the other devices were directly 3D printed.

### Clinical testing

Eleven papers described proof-of-concept studies that did not involve testing of the devices on human participants. Eight of these were studies of endoluminal stents, one was a phantom model, one a scaffold for chemotherapeutic delivery, and one was a robot for ultrasound pain palliation. In the remaining 19 studies which did include human testing, the number of participants ranged from 1 to 92. The most substantial participant groups were recruited in studies of brachytherapy navigation guides (25–92 participants).^38^ The only study that included a control group was of a coplanar navigation guide that was tested on 25 participants.^39^ Ten articles were case reports describing the use of 3D-printed devices for clinical care.

The devices were tested using objective methods in 18 studies, 15 of which produced quantitative results and 2 qualitative. Eight studies used qualitative subjective methods. Two studies used a combination of subjective and objective methods, and two did not report any testing of the device.

### Outcomes of interventions

All reviewed studies reported generally favourable outcomes. Eleven studies confirmed the feasibility of their concept. Nine of these developed endoluminal stents that showed promising results regarding mechanical^18 29^ and drug-eluting properties.^2225^,^27^ It was also reported that such stents could be delivered to patients within 24 hours^18^ or over a weekend at a relatively low cost.^32^ In the other proof-of-concept studies, stent abutment was proven to cause prolonged passage of soft and solid diets^35^; a scaffold for chemotherapeutic delivery was shown to significantly reduce the viability of prostate cancer cells^20^; and MRI safety and compatibility were verified for an ultrasound pain palliation robot.^30^

Anatomical models produced positive outcomes in therapy and surgical planning. They demonstrated a high concordance rate with diagnostic accuracy of invasive procedures,^36^ and facilitated joint-preserving posterior acetabular resection.^11^ In one study, an uncommon anatomical feature was detected that was not recognised in 2D images, but had an important effect on the intraoperative approach.^40^ Head models were produced with satisfactory accuracy to make immobilisation masks without the need for additional patient visits, which lowered treatment costs.^16^

All brachytherapy navigation guides were successfully used, with occasional minor side effects related to the treatment itself. One study included a control group and found significantly higher dosimetry values in target tissues when navigation guides were used.^39^

In general, the fit of patient-specific obturators was satisfactory, and few problems were reported in individual cases (eg, leakage while drinking liquid, nasal voice, numbness, dry mouth).^34^ Patients’ pronunciation, mastication and swallowing were improved, nasal regurgitation was prevented,^19 34^ and the overall psychological and social well-being was enhanced.^34^

Epitheses demonstrated the possibility of improving the patients’ quality of life and comfort, both semifunctionally and aesthetically.^24^ A nasal prosthesis was produced in shorter time and at lower cost compared with traditional techniques.^23^ Endoprostheses for palliative orthopaedic reconstruction were successfully implanted, with significant postoperative pain reduction and improved function of the limb,^28^ and with no cases of poor outcome, severe complications, endoprosthesis failure or migration.^12 28 33 37^ A PEG tube sealing device enabled recommenced feeding regime without leakage within 24 hours from the clinicians’ request.^17^ Finally, the vast majority of patient-specific respirator masks were rated higher than generic masks in all aspects of comfort, leakage, preference, recommendation and tolerance.^31^

When referring to the technology employed, the term ‘3D Printing’ was most often used (25), followed by ‘3DP’ (14), ‘RP’ (12), ‘AM’ (7) and ‘computer-aided manufacturing’ (4).

## Discussion

### The use of 3DP in palliative care

This review identified certain trends in the use of 3DP for the purposes of palliative care. The first study was published in 2004, and two-thirds of the reviewed papers were published after the year 2017. This indicates a considerable increase in the use of 3DP in palliative care in the last few years, which could be directly related to the release/expiration of 3DP patents. Between 2009 and 2014, the original patents for FFF and SLA expired,^41^ leading to the expansion of the 3DP market and subsequent decrease of 3DP entry cost. It is likely that the increase in publications presented in this review is directly related to the democratisation of 3DP. The most prominent fields of application that included clinical testing were radiation oncology (brachytherapy navigation guides) and orthopaedic oncology (anatomical models and endoprostheses). These studies also involved the largest numbers of participants. Brachytherapy navigation guides were among the simplest devices manufactured by 3DP in the included studies, making them relatively easy to implement across a larger number of patients. Anatomical models are relatively easy to make, derived from existing medical imaging, with no ethical constraints or need for regulatory approval, being used for training/education purposes with no body contact, implanting, or any procedure directly impacting the patient. 3DP has been used to manufacture anatomical models dating as far back as the early 1990s,^42^ recently becoming a more familiar and accessible medical application of this technology. Comparably, there have been enough studies to verify 3DP as a go-to technology for endoprostheses and surgical guides. In a review of 3DP techniques in a medical setting in 2016, surgical guides were listed as the most common devices produced (60.0%), followed by anatomical models for surgical planning (38.7%) and implants (12.7%).^43^

### Clinical aspects of 3DP in palliative care

Roughly two-thirds of the reviewed studies reported the outcomes of 3DP-assisted procedures, and one-third were proof-of-concept studies. In general, the clinical outcomes were considered superior to those of conventional approaches. However, only one study (coplanar navigation guide) included a control group that received the treatment without the device.^39^ The lack of a control group can impair the validity of the conclusions drawn, as it is uncertain to what extent clinical results can be attributed solely to the use of the 3D-printed device.

In recent years, 3DP has becoming common practice to treat medical conditions that require highly customised solutions (eg, reconstruction after extensive resection in orthopaedic oncology) and/or high-precision treatment (eg, brachytherapy of unresectable visceral tumours). It can also be used to create devices that do not otherwise exist (eg, a PEG tube sealing device^17^) or are difficult to produce with traditional approaches (eg, obturator for patients with trismus^34^).

### Technological guidelines for 3DP use in palliative care

#### The choice of 3DP technology

In the reviewed studies, 3DP was predominantly used to overcome the difficulties of producing customised devices with traditional methods. FFF was the most commonly used 3DP technology (13 of the reviewed devices, including anatomical models and oesophageal stents). Despite the poor surface finish with an apparent staircase effect typical for low-resolution desktop FFF printers,^23^ it is favoured for its low cost, versatility and wide range of available thermoplastic filaments, allowing clinicians to match material characteristics of the devices with their function. However, in the studies reviewed, there was little evidence of correlation between the type of medical device produced and the choice of 3DP technology, which suggests that 3DP technology was selected based on availability to the clinician rather than its suitability for the specific device. This suggests that some or many 3D-printed medical devices are produced using suboptimal methods due to the lack of funding, accessibility or familiarity with the technology. Table 1 provides a brief overview of the specifications of 3DP technology to inform future clinical practice.^44^,^47^

**Table 1:** Overview of key characteristics of the most common 3DP technologies and materials

	FFF	SLA	DLP	MJ	SLS
Overall cost	Low	Medium	Low	Very high	High
Desktop printers	Yes	Yes	Yes	Yes	Yes
Accuracy	Low	High	High	High	High
Resolution	Low	High	Very high	Very high	Medium
Surface finish	Staircase effect	Smooth	Smooth	Smooth	Grainy
Mechanical properties of printed parts	Satisfactory(anisotropy)	Satisfactory(brittle, affected by moisture and sunlight)	Satisfactory(brittle, affected by moisture and sunlight)	Satisfactory(brittle, affected by moisture and sunlight)	Very good
Complex designs	No	Limited	Limited	Yes	Yes
Multimaterial printing	Yes	No	No	Yes	No
Rigid biocompatible materials— examples	ABS-M30i, PC-ISO,PLA,PMMA,ULTEM^TM^ 1010,ULTEM^TM^ 9085	Accura ClearVue,BioMed Clear,Dental SG Resin,E-Shell 3000,NextDent SG,WaterShed XC 11122	Dental SG Resin,E-Shell 3000	MED610,VeroDent,VisiJet M2R-CL,VisiJet M3 Crystal	CAPA 6501,Duraform PA,EOS PA2200,EOS PEKK,PA 12, PCL
Flexible biocompatible materials—examples	TPU(Tecoflex)	Elastic 50A Resin,E-Guide Soft	E-Guide Soft	MED625FLX,VisiJet M2E-BK70	TPU

ABSAcrylonitrile Butadiene StyreneDLPDigital Light Processing3DPThree-Dimensional PrintingFFFFused Filament FabricationMJMaterial JettingPAPoly Amide (Nylon)PCPolyCarbonatePCLPolyCaproLactonePEKKPolyEtherKetoneKetonePLAPolyLactic AcidPMMAPolyMethyl MethAcrylateSLAStereoLithogrAphySLSSelective Laser SinteringTPUThermoPlastic Urethane

When cost and accessibility are the main concerns, FFF technology is usually opted for, not MJ or SLS. For example, the head mould for radiotherapy immobilisation mask would be too expensive to manufacture using other technologies, and the proof-of-concept studies of stents used FFF possibly due to accessibility for research purposes. For devices in direct contact with the skin or mucosa, such as obturators, smooth surface finish is often important, and thus, SLA, DLP or MJ are favoured. Likewise, the surface finish of epitheses should resemble the texture of skin, which cannot be achieved with FFF, as pointed out in a study of a nasal epithesis.^23^ Similar to surface finish, FFF would be rejected for accuracy and resolution in place of MJ, DLP, SLA or SLS, especially when producing highly detailed parts, such as the thread of a Percutaneous Endoscopic Gastrostomy (PEG) tube sealing device or implants.

#### The choice of 3DP materials

Half of the reviewed papers did not detail the material employed. Across the other studies, the most common material, ABS, is used largely for moulds for its high strength, toughness and impact resistance, flexibility, durability and temperature resistance which allows for mould reusability.^23 47 48^ For other FFF applications, PLA can be favoured over ABS due to its ease of printing, accessibility and price.^16^ PCL is used to manufacture endoluminal stents because of its biocompatibility and bioresorbability.^26^ Similarly, biocompatibility is the reason for using MED610 for devices that are expected to stay in prolonged contact with the patient’s skin.^17^ Endoprostheses for palliative purposes can stray from the typical use of titanium alloys,^33 37^ as integration between the host bone and endoprosthesis is not expected in patients with bone metastases. In this case, PMMA can be employed as an alternative 3D-printable biocompatible material that is generally available and sufficiently strong to replace non-weight bearing bone, while also being more cost-efficient.^12 49^

Navigation guides for brachytherapy should be safe for skin contact, and are mainly fabricated from photopolymer resins. A common issue with photopolymer resins is the cytotoxicity of the raw material, therefore, a careful balance in its composition is required to preserve printability and ensure safety for use.^50^ Among the most versatile biocompatible polymers used with photo-curing techniques are acrylate- and methacrylate-based resins.^47^

#### Manufacturing approaches

Patient-specific devices are reverse engineered by using digital data of patients’ anatomy, as opposed to being directly designed. Indirect AM can be used to create moulds for devices that need to be manufactured from non-printable materials, e.g. silicone, like obturators and epitheses. 3DP materials approved for human use with similar properties to silicone are scarce, and most biocompatible silicone resins are not yet commercially available.^50^ Among those currently on the market, 3D-Bioplotter UV Silicone 60 A MG (EnvisionTEC) is a transparent medical-grade silicone, approved for 29-day direct skin contact, characterised by medium hardness, no odour, and the possibility of colouring prior to printing.^51^ Similarly, TrueSilTM (Spectroplast AG) is biocompatible and available in different hardnesses for different applications (eg, mouthpieces, insoles, earbuds, prosthetics).^52^ Elastic Resin (Formlabs) mimics casted silicone well, but it is not biocompatible.^53^

### Regulatory aspects of 3DP in medicine

Currently, 3D-printed medical devices must conform to the same regulations as those that are manufactured using traditional methods. The regulations vary across different countries (eg, Regulation (European Union, EU) 2017/745 on Medical Devices Reporting^54^ in the EU; Title 21 Code of Federal Regulations^55^ in the USA), and have been extensively reviewed in other literature.^56 57^ The standard approval process for new medical devices tends to be lengthy, requiring several years of preclinical and clinical testing. As this can present a substantial barrier to urgently treating rare, life-threatening or severely debilitating medical conditions, not uncommon in palliative care, non-standard regulatory pathways have been established for rapid approval of medical devices in exceptional circumstances. These pathways allow for clinicians and/or manufacturers to apply for exemptions to use non-certified medical devices on humanitarian grounds. The use must be justified through a significant reduction in mortality or morbidity compared with alternative compliant treatments, and applications are assessed on a case-by-case basis.

The vast majority of studies included in the present review did not detail the regulatory frameworks followed. An overview of regulatory aspects applicable is provided the authors’ previous systematic review of 3D-printed medical devices used on patients^3^. Especially when bespoke medical devices are 3D-printed to be used without prior testing under the above-mentioned humanitarian exemptions, it is of utmost importance that an appropriate quality management system is in place, which can ensure that appropriate technologies and materials (eg, certified biocompatible materials) are employed in the printing process, and that the postprocessing requirements are met to warrant mechanical, chemical and biological safety of the end product.^58^

### 3DP and design collaboration

This systematic review highlights how 3DP can potentially be used as part of a design process to address previously unmet clinical needs for which current solutions are either not available or not suitable. The majority of the studies indicated authorships which were interdisciplinary, typically between clinical and design/technical groups. The papers typically focused on the clinical problems and the reporting of the solutions obtained, and therefore, it is not possible to ascertain and synthetise the desgin processes followed across the studies. The current authors anecdotal experience is that clinicians sometimes issue requests to research groups in universities for design assistance with very specific clinical challenges. Arising from these requests, clinical design collaborations are initiated which often form the basis of followon 3DP/innovation research. By way of example, we previously reported on a clincal request to our group for assistance to produce an alternative eye cover for a teen with Rhabdomyoscarcoma.^59^ Access to3DP was not part of the initial request but was used by the design group to make the solution. Arising from the engagement, the local palliative care clinical team and the design group thereafter established other research opportunites regarding 3DP in palliative care. Hence, once initial experience is established, then followon design interactions using 3DP are made possible.

Our experience is that some clinicians have experience in 3DP, either through previous clinical innovations or due to access to promoted clinical-based 3DP programmes. In these situations, such clinicians may develop their own concepts for which their key requirement thereafter is access to designers to collaborate in refining the design and print the concepts/devices.

### Limitations

There may be other studies not identified by our systematic search due to the terminology issues addressed above, thus, it is possible that some 3D-printed devices intended for palliative care were not included in this review. Moreover, the identified cases of palliative correction of congenital heart defects typically managed by cardiologists were excluded. Nevertheless, the authors expect the key findings of the present work to be a reasonably complete reflection of the current state regarding the use and potential for increased use of 3DP in the provision of care to patients with palliative care needs.

## Conclusions

This systematic review revealed the use of 3DP in palliative care for approximately two decades, with a considerable increase in its use since 2017. Reviewed were 36 devices produced across 30 studies. The device type, field of application, problem addressed, technology used, clinical testing methods and the outcomes of intervention were analysed.

The most common proof-of-concept devices were endoluminal stents, and the most common devices that included clinical testing were anatomical models, brachytherapy navigation guides and endoprostheses. Of the 3DP technologies, FFF was most frequently employed, followed by SLA and MJ. In most of the studies that specified the material used, ABS was chosen, mainly for creating moulds, followed by unspecified photopolymer resins. The majority of devices were designed using RE to correspond to the patient’s anatomy. The outcomes of interventions were generally favourable, and the difficulties associated with conventional procedures were successfully overcome. 3DP was found especially valuable in the treatment of medical conditions that require highly customised solutions and/or high-precision procedures, while also ensuring cost-efficiency and time-efficiency. With 3DP, entirely new devices can also be created for rapid response to unique clinical situations.

## supplementary material

10.1136/bmjspcare-2021-003196 online supplemental file 1

## Data Availability

All data relevant to the study are included in the article or uploaded as online supplemental information.
