# Supplementary material for: 3D printing in palliative medicine: systematic review
Source: BMJ Support Palliat Care. 2022 Sep 6;14(e3):e003196. doi: 10.1136/bmjspcare-2021-003196 (PMC11671886; doi:10.1136/bmjspcare-2021-003196)
Supplement: online supplemental file 1 [file bmjspcare-14-e3-s001.pdf]

Supplemental table Reviewed studies of 3DP in palliative care.

Study **Field of application**<br>Device type and indication	**Problem solved by 3DP**	**Device Production**		**Device testing/use**		
		**Imaging technique**<br>Software	**3DP technology**<br>3D printer (manufacturer)<br>Material<br>*Additional procedures (if applicable)*	**Participants**<br>Number, sex, age<br>Medical status	**Methods**	**Outcomes**
**[35] Gastrointestinal oncology (gastroduodenal)**<br>Anatomical phantom for testing of gastroduodenal stents for treatment of malignant strictures	Ethical unacceptability of investigating the mechanism and significance of stent abutment in the duodenal wall of live patients.	**CT**<br>MeshLab<br>Meshmixer	**MJ**<br>Objet500 Connex3 (Stratasys)<br>Tango family	<u>Retrospective analysis</u><br>♂, 62 years<br>Advanced gastric cancer	Measurement of elapsed times at passage of water (300 ml, 4 s), and soft and solid food materials (3 types; 300 ml); partially and fully covered self-expanding metallic stent; 2 locations of distal stent ends; 10 repetitions.	Proof of concept: Stent abutment can cause prolonged passage of soft and solid diets through the stent, impaction of solid diets into stent.
**[36] Gastrointestinal oncology (bile duct)**<br>Patient-specific anatomical model of tumor and bile duct visualization to aid surgical planning for ERCP biliary stent placement	Difficulty determining target bile duct with traditional imaging techniques in complex HCC.	**CT/MR**<br>Mimics 17.0	**MJ**<br>ProJet 4500 (3D Systems)<br>VisiJet C4 Spectrum Core	<u>Retrospective analysis</u><br>6♀, 9♂, 65.4 ± 14.9 years<br>Inoperable hilar cholangiocarcinoma	Target bile duct and Bismuth-Corlette (BC) classification on the basis of 3D models; comparison with those in ERCP.	86.7% concordance rate of target bile duct, 93.3% concordance rate of BC type classification with 3D model compared to ERCP.
**[11] Orthopaedic oncology (pelvis)**<br>Patient-specific anatomical model of the pelvis for surgical planning to minimize acetabular bone loss and maximally preserve native hip function and stability	Difficulty of safe tumor resection with negative oncological margins, acceptable postoperative function, preservation of critical neurovascular structures and minimal perioperative morbidity, mortality and recurrence within tightly confined, complex anatomical areas.	**CT**<br>N/A	**N/A**<br>N/A<br>N/A<br>Manufactured by Onkos Surgical	♂, 21 years<br>Metastatic osteoblastic osteosarcoma	Clinical follow-up.	Successful joint-preserving posterior acetabular resection of metastatic osteosarcoma with tumor-free margins and preserved hip stability. Improved quality of life, patient returned to athletic and academic pursuits.
**[16] Radiation oncology (brain)**<br>Patient-specific anatomical model of the head and head-and-neck rest to serve as a volume and position mould for radiotherapy immobilization mask in whole brain radiotherapy	The need for an additional simulation CT-scan in preparation for radiotherapy with traditional methods, which increases the number of patient visits, interventions and waiting times.	**CT**<br>CATIA	<u>Mould:</u><br>**FFF**<br>BigBuilder Dual-Feed (Builder 3D Printers BV)<br>PLA<br>*<u>Immobilization mask:</u>*<br>*Thermoplast (Aquaplast RT) moulded onto model head*	<u>Retrospective analysis</u><br>9♀, 2♂, 60 ± 11 years (range 47-85)<br>Brain metastases	CT scan of immobilization mask and comparison of volume to patient CT scan in Eclipse. Calculation of simulated radiation dose and comparison.	98.1% similarity between patient head surface geometry and 3D model (model volume 1.6% smaller due to segmentation smoothing), reproduction accuracy for head position within institutional constraints. Minimal differences in dosimetry during whole brain radiotherapy. Lower cost compared to simulation CT-scan, potential for reducing patient visits and waiting times.

**[40] Gastrointestinal oncology (pancreas)**<br>Patient-specific anatomical model for pylorus-preserving pancreatic head resection and reconstruction planning in locally advanced adenocarcinoma	Difficulty establishing detailed anatomy from 2D CT images, especially in complex and unconventional cases.	**CT-angiography**<br>N/A	**N/A**<br>N/A<br>N/A (multimaterial)	♂, 71 years<br>Locally advanced adenocarcinoma of the papilla vateri; metastatic squamous-cell carcinoma of the lung (4 years stable); previous right hemicolectomy and patch plasty of the celiac trunk and superior mesenteric artery; primary adrenal insufficiency; Bühler anastomosis	/	Successful tumor resection. Bühler anastomosis only detected in 3D reconstruction; perioperative anatomy visualization using 3DP has the potential to increase patient safety.
**[29] Gastrointestinal oncology (oesophagus)**<br>Self-expanding plastic oesophageal stent to alleviate the symptoms of irresectable oesophageal malignancies	Traditional manufacturing methods do not enable time-efficient production of parts with custom geometry and structure.	**/**<br>Multiphysics™	**FFF**<br>Ultimaker 2 (Ultimaker)<br>PLA/TPU composite (0:100, 5:95, 10:90, 15:85)	/	In silico, in vitro and ex vivo evaluation: Finite element analysis, testing of self-expanding properties, compression forces, self-expansion and anti-migration forces (porcine oesophagus), 16-week hydrolytic degradation rate (phosphate buffered saline, simulated gastric fluid), biocompatibility test (human primary oesophageal epithelial cells).	Proof of concept: Significantly higher anti-migration force compared to existing stents, reduced migration distance, adjustable self-expansion force.
**[25] Gastrointestinal oncology (oesophagus)**<br>Patient-specific oesophageal endoluminal drug-eluting stent for sustained local delivery of 5-FU to achieve short-term reduction of tumor size in patients with oesophageal cancer	Wide morphological and clinical variability of gastrointestinal tumours can affect the performance of non-customisable drug-eluting stents.	**/**<br>SolidWorks<br>Cura	**FFF (dual extrusion)**<br>Ultimaker S5 (Ultimaker)<br>PU	/	Material, mechanical and in vitro evaluation: Analysis of 5-FU distribution (photoacoustic Fourier-transform infrared spectroscopy), topography (scanning electron microscopy), mechanical properties (local compressive force, recovery rate), thermal analysis, drug content (high-performance liquid chromatography), in vitro drug release over 110 days, 5-FU stability following stent sterilization (UV, gamma irradiation) and accelerated storage (different temperatures and humidities)	Proof of concept: Confirmed homogeneous dispersion of 5-FU throughout the PU matrix, sustained release profile over 110 days, permeability from stent through oesophageal tissues, negligible degradation during thermal processing, minimal degradation during sterilization, reasonable stability over 3 months of accelerated storage.
**[26] Gastrointestinal oncology (oesophagus)**<br>Tissue-specific EdECM hydrogel-loaded oesophageal stent to alleviate symptoms of radiation esophagitis	Limited precision and architectural control in hydrogel-loaded stent fabrication using traditional technologies (e.g. braiding, knitting, laser-cutting, segmentation).	**/**<br>N/A	**FFF (spindle)**<br>2RPS (custom)<br>PCL	/	Material, mechanical, in vitro evaluation, and in vivo animal study: Stent surface morphology (SEM) and topography (AFM) analysis, static compression test to evaluate radial forces, cyclic 3-point bending to test flexibility and mechanical stability; rheological assessment of EdECM hydrogel, viability assessment of human oesophageal Het-1A cells in EdECM; radiation esophagitis rat model for evaluation of therapeutic effects.	Proof of concept: Therapeutic effects confirmed on animal model: resolved inflammatory response, facilitated tissue regeneration. Promising clinical approach to local delivering of therapeutic cells/drugs to manage disease.

**[22] Gastrointestinal oncology (bile duct)**<br>Patient-specific biliary stent with stem cell-collagen-cholangiocyte coating to provide relief from malignant and benign bile-duct obstructions	Progressive loss of biliary stent patency over time due to biofilm and biliary 'sludge' formation.	/<br>TinkerCAD<br>ImageJ<br>Makerbot	<u>Stent:</u><br>**FFF**<br>Replicator (Makerbot)<br>PVA (Aquasolve)<br><u>Collagen injection moulding chamber:</u><br>**SLA**<br>Form 2 (Formlabs)<br>Flexible Resin<br>*Stem cell collagen injection moulding, stent maturation, cholangiocyte seeding*	/	Material and in vitro evaluation: Stent surface morphology (X-ray), human placental mesenchymal stem cell and cholangiocyte viability assessment (high-resolution Cryo-SEM, phase microscopy, flow cytometry, immunofluorescent imaging)	Proof of concept: Successful incorporation of cholangiocytes to improve stent patency by reducing the entrance and adherence of harmful bacteria.
**[27] Gastrointestinal oncology (bile duct)**<br>Self-expanding, drug-eluting biliary stent for palliative treatment of biliary obstruction in unresectable hilar malignancies	Limited architectural, dosage precision, drug distribution and release control in drug-loaded stent fabrication using traditional technologies.	/<br>N/A	**FFF**<br>N/A<br>PCL/PTX	/	Material, mechanical, and in vitro evaluation: Surface morphology (optical microscope, FE-SEM), radial and axial forces, chemical and thermal structure, degradable behaviour and drug release (porcine bile solution, 8 weeks), inhibitory effect on tumor growth (human biliary tract cancer cells, nude mice).	Proof of concept: Confirmed uniform drug distribution and steady release in vitro, no changes in weight and shape over time, inhibitory effect on tumor cell proliferation in small animals.
**[32] Pulmonary oncology**<br>Stent master for silicone moulds to rapidly produce customised airway stents for treatment of life-threatening tracheobronchial obstructions in patients with respiratory cancer	Long manufacturing times and high costs of conventional manufacturing methods, short durability of silicone moulds.	/<br>N/A	**SLA**<br>N/A<br>N/A	/	Testing of airway stent customisation protocol: estimation of time required to deliver customised stent to the patient.	Proof of concept: Possibility of providing relief to patients within a day or over the weekend, at a relatively low cost.
**[18] Pulmonary oncology**<br>Customised tracheobronchial stent to provide relief in respiratory tract obstruction by tumours or other lesions	Need for rapid airway-stent customisation in unusual airway morphology or unresectable, stiffer than normal lesions to suit the airway geometrical and distending strength requirements for effective palliation.	/<br>N/A	<u>Stent master:</u><br>**SLA**<br>N/A<br>N/A<br>*<u>Mould and final stent:</u>*<br>*Casting of silicone to create stent mould, vacuum casting of PU-based resin for final stent*	/	Mechanical testing: distending strength, collapsibility; comparison to Dumon stent.	Proof of concept: Distending strength comparable to, collapsibility 17% larger than Dumon stent. The stent could be delivered to the patient in 24 h.
**[18] Gastrointestinal oncology (colon)**<br>Customised colorectal stent to provide relief in occlusion by colorectal cancer	22-23% mortality rate of surgical procedure to create temporary stoma before resection of the stricture. Existing colonic stents are costly and nonreusable.	/<br>N/A	<u>Stent master:</u><br>**SLS**<br>N/A<br>N/A<br>*<u>Mould and final stent:</u>*<br>*Casting of RTV9 silicone to create stent mould, vacuum casting of PU-based resin for final stent*	/	Mechanical testing: distending strength, collapsibility.	Proof of concept: Superior collapsibility ratio to conventional polymer stents, surpassed required collapsibility required for effective irrigation of the bowel. Comparable strength to metal colonic stents (e.g. ChooStent).
**[21] Gastrointestinal oncology (oesophagus)**<br>Master for vacuum casting of semi-rigid and rigid auxetic oesophageal stents for palliative treatment of oesophageal cancer and prevention of dysphagia	N/A	/<br>Inventor	<u>Stent master:</u><br>**FFF**<br>N/A<br>ABS<br>*<u>Stent:</u>*<br>*Vacuum casting of PU resin (PX 212, VC-3300)*	/	Surface characterization (SEM), mechanical characterization (tensile and expansion testing), finite element analysis	Proof of concept: Radial expansion 0.5-5.73 mm, longitudinal extension 0.15-1.83 mm at applied pressures 0.5–2.7 bar from balloon catheter. Possibly good conformation to oesophageal wall due to non-linear anisotropic mechanical response.

**[60] Radiation oncology (brachytherapy rectum)** Patient-specific non-coplanar navigation guide for RIS implantation in palliative treatment of locally recurrent rectal cancer	Efficiency and accuracy of RIS implantation using traditional approaches relies on operators' experience, and misplacement can lead to unsatisfactory outcomes.	**CT** N/A	**N/A** N/A Photopolymer resin	28♀, 38♂, median 56 years (range 32-79) Recurrent sacral-invasive, lateral-invasive, or localized rectal cancer; post chemotherapy, EBRT, or surgical resection	Post-operative dose evaluation (CT); Clinical evaluation at follow-up (2.5-35.9 months): blood test, tumor markers test, abdominal and chest CT, pelvic MR imaging, tumor response (RECIST guideline version 1.1), pain assessment (Numeric Rating Scale), side-effect evaluation (toxicity criteria of the Radiation Therapy Oncology Group), overall survival time.	Confirmed effectiveness and safety of salvage treatment strategy: 85.1% pain relief, 9.1% severe side effects, median overall survival time 14.7 months, median local control time 12.2 months.
**[61] Radiation oncology (brachytherapy head and neck)** Non-coplanar navigation guide for RIS implantation in palliative treatment of recurrent malignant head and neck tumours	Limited accuracy of RIS implantation using traditional approaches.	**CT** N/A	**N/A** N/A Photopolymer resin	14♀, 28♂, median 61 years (range 29-79) Recurrent or metastatic head/neck tumor: naso-/hypo-/oropharyngeal, oral, laryngeal, salivary-gland, thyroid, oesophageal, cervical, lung, breast, or colon cancer, soft-tissue sarcoma, lymph-node metastasis of unknown aetiology	Post-operative dose evaluation (CT); Clinical evaluation of side effects at follow-up (4-14 months): skin puncture: bleeding, pain, infection, non-union of puncture point, metastasis due to RIS implantation; radiation (toxicity criteria of the Radiation Therapy Oncology Group and the European Organization for Research and Treatment of Cancer): skin injury, mucosal response, spinal-cord injury, peripheral-nerve injury, xerostomia, blood toxicity; nerve injury (Common Terminology Criteria for Adverse Events v4.0); seed migration.	Successful RIS implantation with good accuracy of positioning. 3 cases of grade 1 acute skin reaction, no cases of grade >3 reactions. No blood toxicity, no spinal cord injury, 1 case of grade 3 nerve response.
**[62] Radiation oncology (brachytherapy various)** Patient-specific navigation guide for CT-guided RIS implantation in treatment of advanced malignant tumours	Limited accuracy of RIS implantation, and associated unwanted effects using traditional approaches.	**CT** N/A	**N/A** N/A Medical resin	18♀, 24♂, 58.9 ± 14.1 years (range 25-91)	Patients' quality-of-life assessment: EORTC QLQ-C30 (4-point scale: 1 - not at all, 4 - very much): function (physical, role, cognitive, emotional, social), symptoms (fatigue, pain, nausea/vomiting), single measurement items, global quality of life; administered prior to surgery, at 24 h, 1 and 3 months after surgery.	Average EORTCQLQ-C30 score after seed implantation higher at 1-month follow-up compared to 24-hour and 3-month follow-up.
**[39] Radiation oncology (brachytherapy pancreas)** Coplanar navigation guide for RIS implantation in treatment of pancreatic cancer	Limited accuracy and considerable complexity of RIS implantation using traditional approaches; impossible real-time adjustment of puncture direction with non-coplanar guides.	**/** N/A	**N/A** N/A PMMA	Experimental group: 6♀, 6♂, median 65.5 years (range 48-81) Control group: 7♀, 6♂, median 63.8 years (range 47-84) Unresectable pancreatic carcinoma	Between-group comparison of post-operative dose (CT), implementation success rate and complications.	Successful RIS implantation without major complications; 1 self-limiting, clinically insignificant local hematoma due to mesentery vessel injury. Dosimetry values significantly higher in experimental compared to control group.
**[38] Radiation oncology (brachytherapy rectum)** Patient-specific non-coplanar navigation guide for RIS implantation in palliative treatment of primary or metastatic thoracic tumours	Discrepancy between the postoperative target dose and preoperative plan in freehand RIS implantation.	**CT** N/A	**N/A** N/A Medical curing resin	32♀, 60♂, median 62 years (range 17-88) Primary or metastatic solid, unresectable malignant tumor of the lung, chest wall, or mediastinum	Clinical examination and assessment of toxicity effects (radiation pneumonia, esophagitis, skin reaction, myelitis, cardiotoxicity) at follow-up (median 10.7 months). Overall survival and local control duration and rate at 1 and 3 years.	Toxicity effects: 3 grade ≥2 radiation pneumonia, 2 grade ≥2 radiation esophagitis, 1 oesophageal fistula, 2 tracheal fistulae, 1 chest-wall pain, 3 haemoptysis, 5 grade 2 radiation skin reaction; no defined radiation myelitis or cardiotoxicity. 34 cases of pneumothorax. Overall survival: median 15 months; 59.7% (1 year), 22.2% (3 years). Local control: median 16.4 months; 64.9% (1 year), 32.8% (3 years); significantly better for metastatic than primary cancer.

**[19] Maxillofacial oncology**<br>Definitive cast for manual fabrication of the mould to produce a patient-specific self-retentive interim obturator for palliative palate reconstruction after partial maxillectomy due to cancer	Inability to fabricate the obturator using impression trays due to limited maximal incisal opening.	**CT**<br>Mimics<br>SpaceClaim	Definitive cast:<br>**MJ**<br>Objet260 Connex 3 (Stratasys)<br>ABS (RGB 515 Digital ABS)<br>*Duplicate definitive cast:*<br>*PolyPour mould, type III dental stone cast*<br>*Obturator:*<br>*Wax positive mould, 3-piece dental stone negative mould, packing of platinum silicone elastomer (A-RTV-40), colouring, trimming, contouring*	♀, 55 years<br>Post unilateral maxillary resection due to T4aN0M0 invasive squamous cell carcinoma with bone and perineural invasion, failed free-flap reconstruction, severe trismus	Visual inspection of fit, patient feedback regarding comfort	Restored functional quality of life: improved speech and mastication, prevented nasal regurgitation.<br>Eliminated need for impression materials.
**[34] Maxillofacial oncology**<br>Definitive cast for manual fabrication of the mould to produce a patient-specific obturator for palliative palate reconstruction after partial maxillectomy due to cancer	Labour-intensive, time-consuming fabrication of one-piece obturators using traditional techniques because of access limitations due to soft tissue fibrosis and trismus after maxillectomy and radiation.	**CT**<br>Mimics<br>Geomagics Studio 12	Definitive cast:<br>**MJ**<br>ProJet HD 3500 (3D Systems)<br>N/A	4♀, 7♂, 44 ± 16 years (range 25-68)<br>Post partial maxillectomy due to squamous cell, mucoepidermoid or adenoid cystic carcinoma, myofibroblast or fibromyxoid sarcoma	Assessment of obturator retention, marginal fit and occlusion, and excessive tissue displacement. Patient feedback at 1-week follow-up (Obturator Functioning Scale of the Memorial Sloan-Kettering Cancer Center): problems with eating, speech difficulties, dry mouth, poor aesthetics, upper lip paraesthesia, difficulty inserting the obturator, avoidance of routine social interactions); 3-point scale: 1 - not at all/a little, 3 - very much/extremely.	Maximum support, stability and retention; 1 case of leakage drinking liquid, no leakage while swallowing; 2 cases of somewhat nasal voice; 3 cases of clasps noticeable on anterior teeth; 3 cases of extreme numbness; 3 cases of dry mouth. Overall, regained functions of mastication, pronunciation and swallowing, improved psychological and social wellbeing.
**[24] Maxillofacial oncology (facial epithesis)**<br>Patient-specific working model and mould for facial prosthesis to restore the cosmetic appearance of patients with facial defect after ablative tumor surgery	High cost of customised products, and difficulty producing geometrically complex parts with traditional technologies.	**3D Scanner HD "Ear & Nose Digital Library"**<br>ScanStudio<br>RapidForm	Working model and mould:<br>**FFF**<br>Dimension (Stratasys)<br>ABS P400<br>*Final epithesis:*<br>*Silicone casting, colouring*	♀, 73 years<br>Recurrent squamous-cell carcinoma, infiltrating facial bone	N/A	Possible improvement of patients' quality of life and comfort, semi-functionally and aesthetically.
**[23] Maxillofacial oncology (nasal epithesis)**<br>Mould for patient-specific nasal prosthesis to restore the cosmetic appearance of patients with facial defect after ablative tumor surgery	High cost and difficulty producing geometrically complex parts with traditional technologies, especially in the absence of symmetric body part to mirror.	**3D Scanner HD "Ear & Nose Digital Library"**<br>ScanStudio<br>RapidForm	Mould:<br>**FFF**<br>N/A (Stratasys)<br>ABS P400<br>*Final epithesis:*<br>*Silicone casting, colouring*	♂, 67 years<br>Post ablative surgery of the nose due to recurrent squamous-cell carcinoma, failed plastic reconstruction	Analysis of production cost and time, comparison with traditional techniques.	Reduced time and cost of the procedure compared to traditional techniques.
**[12] Orthopaedic oncology (upper extremity)**<br>Patient-specific endoprosthesis for palliative upper-extremity reconstruction after bone-metastasis resection	Challenging production, time- and cost-inefficiency of traditional methods for producing patient-specific endoprostheses for reconstruction after extensive bone resection.	**CT**<br>Mimics	**Selective Laser Lithography***<br>N/A (MTEC)<br>PMMA	16 patients<br>Humeral, ulnar metastases of multiple myeloma, prostate, breast, lung adenocarcinoma, thyroid follicular-cell carcinoma, cholangiocarcinoma, or osteosarcoma	Functional outcome evaluation: MTSS (5-point scale), Mankin score (poor-good); elbow and forearm range of motion assessment; Endoprosthesis failure evaluation: prosthesis fracture, prosthesis-bone interface failure, prosthesis and fixative device failure at follow-up (38-1324 days).	Production of affected-bone replicas within 48 h. Successful reconstruction with good shoulder stability, low rate of complication, and no cases of poor outcome or endoprosthesis failure. 3 of 13 cases of soft tissue failure in first 6 months. Mean MTSS 55%, higher for emotional acceptance and associated pain compared to functional activities, lifting ability, and hand positioning; Mankin score: 64% good, 36% fair.

**[37] Orthopaedic oncology (pelvis)**<br>Patient-specific pubic bone endoprosthesis for pelvic ring reconstruction after exenteration for anal cancer recurrence	Poor physical well-being and postoperative quality of life after complete pelvic exenteration without pubic symphysis reconstruction when osteosynthesis is not possible.	**CT/MR**<br>Mimics 21.0	**DMLM**<br>Concept Laser M2 cusing (GE Additive)<br>Rematitan (90% titanium, 6% aluminium, 4% vanadium)<br>Designed by TIOS, Ltd. (Moscow, Russia)	♀, 52 years<br>Recurrent anal squamous cell carcinoma with vagina, bladder, and pubic bone invasion, locoregional failure after chemotherapy	SF-36: qualitative assessment of patients' perceptions of their physical functioning, physical and emotional limitations, social functioning, bodily pain, general and mental health.	Successful endoprosthesis implantation 5 weeks after initial pelvic exenteration (R0 resection), without severe complications. Patient started to walk 9 days and resumed normal activities 6 weeks after surgery. No recurrence or implant rejection at 6-month follow-up. SF-36v2: 62% with positive dynamics in physical health status.
**[33] Orthopaedic oncology (pelvis)**<br>Patient-specific osteotomy guide and hemi-pelvic endoprosthesis for metastatic acetabular carcinoma resection and pelvic reconstruction	High incidence of prosthetic mismatch and loosening in pelvic reconstruction surgery with conventional modular prostheses.	**CT**<br>Mimics 17.0<br>Magics 18.0	<u>Pelvic model:</u><br>**SLA**<br>iSLA-450 (Shining 3D)<br>N/A<br><u>Osteotomy guide:</u><br>**SLS**<br>Formiga P110 (EOS)<br>N/A<br><u>Endoprosthesis:</u><br>**EBM**<br>Arcam A1 (Arcam Corporation)<br>Ti6Al4V	♀, 62 years<br>Destructive metastatic acetabulum carcinoma, right renal clear-cell carcinoma	Clinical assessment preoperatively and at 12-month follow-up: MSTS score: rating (0-5 scale) of pain, function, emotional acceptance, use of external support, walking ability, gait alteration; Harris Hip Score: rating (1-4 scale) of pain, support, walking distance, limp, activities, sitting, stair climbing, public transportation; RoM; SF-36: qualitative assessment of patients' perceptions of their physical functioning, physical and emotional limitations, social functioning, bodily pain, general and mental health.	Accurate tumor resection and pelvic reconstruction without prosthetic loosening or migration, periprosthetic fractures, or infection; walking 1 km with assistance at 6 months, independent at daily activities at 12-month follow-up. Improved MSTS result (from 7 to 16), SF-36 (from 52.7 to 108.2), and Harris score (from 19 to 52).
**[28] Orthopaedic oncology (upper limb)**<br>Patient-specific mould for bone-substitute endoprosthesis to reconstruct upper-limb long bones after tumor resection	Limited possibilities of traditional methods to produce custom shape, size and complexity of endoprostheses in large limb-segment lesions.	**CT**<br>N/A	<u>Mould:</u><br>**FFF**<br>Replicator 2 (MakerBot)<br>N/A<br><u>*Endoprosthesis:*</u><br>*Casting of bone substitute material (PMMA or Rekost) with allograft chips*	12♀, 10♂, 50.9 ± 7.2 years (range 41-62)<br>Lesion at the humerus, ulna or radius due to enchondroma, osteoblastoclastoma, non-osteogenic fibrosis, solitary bone cysts, osteogenic sarcoma, metastasis of mammary or prostate gland and lung cancer.	Clinical assessment preoperatively and at 12-month follow-up: SF-36: qualitative assessment of patients' perceptions of their physical functioning, physical and emotional limitations, social functioning, bodily pain, general and mental health; MSTS score: rating (0-5 scale) of pain, function, emotional acceptance, use of external support, walking ability, gait alteration; VAS: pain assessment.	Significant postoperative pain reduction and improved function of the upper limb. Benign cases: SF-36: 71.4 ± 6.6, VAS: 2.5 ± 1.5, MSTS: 65.1% ± 8.3%; malignant cases: SF-36: 39.2 ± 4.3, VAS: 4.8 ± 1.4, MSTS: 41.8% ± 5.2%. No statistically significant differences depending on material used. No radiographic evidence of implant migration, 4 cases of marginal osseointegration with Rekost.
**[17] Enteral feeding**<br>Bespoke sealing device for emergency repair of leaking PEG tubes in patients ineligible for surgical replacement procedure	Disrupted enteral feeding in patient ineligible for surgical PEG-tube replacement, significant risks associated with non-operative methods of replacement, no existing repair kits	**/**<br>SolidWorks	**MJ**<br>Connex500 (Stratasys)<br>MED610	♂, 15 years<br>Advanced cystic fibrosis	Application of sealing device, check for leaks at tube flushing.	PEG-tube function restored within 24 h from first contact between medical staff and designers. PEG tube ready for use immediately after application of sealing device, patient recommenced on feeding regime.
**[31] Neurology (ALS)**<br>Patient-specific interface for a generic mask for noninvasive ventilator support in chronic medical conditions with weakness of respiratory muscles	Generic masks in standard sizes are often uncomfortable, ill-fitting, and leaky, which causes patients to discontinue using them.	**MR** (patient's face)<br>Mimics<br>**Vivid 9i (Konica Minolta)** (modified oronasal mask Veraseal 2)<br>Visualization toolkit library<br>Blender 2.78	<u>Rigid interface,</u><br><u>mould for silicone interface:</u><br>**SLA**<br>Form 2 (Formlabs)<br>Standard clear resin<br><u>*Silicone interface:*</u><br>*Casting of Dragonskin10 (Smooth-On)*	♂, age N/A<br>Variant ALS, unique facial contours (high nose bridge, lower-jaw overbite), prescription glasses	1-night trials with 10 modified masks and 2 standard masks; two 7-night trials with favourite mask. Custom questionnaire for patient evaluation of comfort, leakage, preference, recommendation, tolerance (Likert-type 1-5 scale questions), open-ended questions.	Scores for favourite masks over 7-night trials: comfort: 4, 4; leakage: 3, 2; preference: 4, 3; recommendation: 4, 3; tolerance: 4, 3. Compared to standard mask, 8 of 10 custom masks were rated higher in all aspects. Common complaints: masks were unable to accommodate his sleep movement and jaw movement.

**[20] Oncology – Chemotherapy (spine)**<br>Nanoporous scaffold for local chemotherapeutic delivery in spine metastases of prostate cancer	Severe side effects of high systemic doses of chemotherapeutics; large defects after surgical removal of spine metastases that cannot spontaneously heal and require bone grafting.	/<br>SketchUp<br>Simplify 3D	**FFF**<br>Creator Pro (Flashforge Corp.)<br>TPU/PVA (PORO-LAY series: Lay FOMM 60, 40; Gel Lay)<br>*Doxorubicin loading on scaffolds*	/	Assessment of doxorubicin release from scaffolds over 7 days (fluorescence detection); tumor-cell metabolic activity and proliferation assessment; scaffold porosity evaluation (SEM).	Proof of concept: 60-75% of doxorubicin loaded onto scaffolds was released over 7 days, significantly reducing metabolic activity and proliferation of prostate cancer cells and spine metastases cells.
**[30] Oncology – Pain palliation (bone)**<br>Portable MRI-guided robotic system for pain palliation in bone cancer using thermal ablation with focused ultrasound	N/A	/<br>N/A	**FFF**<br>FDM400 (Stratasys)<br>ABS	/	Evaluation of robot functionality (gel phantoms), and MRI safety and compatibility.	Proof of concept: Verified MRI safety and compatibility, and the capacity for creating discrete and multiple overlapping lesions in a gel phantom.

5-FU – 5-fluorouracil, ABS – Acrylonitrile Butadiene Styrene, AFM – Atomic Force Microscopy, BJ – Binder Jetting, CT – Computed Tomography, DMLM – Direct Metal Laser Melting, EBRT – External Beam Radiotherapy, EdECM – Esophagus-derived decellularized Extracellular Matrix, EORTC QLQ-C30 – European Organization for Research on Treatment of Cancer Quality of Life Questionnaire, ERCP – Endoscopic Retrograde Cholangiopancreatography, FE-SEM – Field Emission-Scanning Electron Microscopy, FFF – Fused Filament Fabrication (also FDM – Fused Deposition Modelling), MJ – Material Jetting, MR – Magnetic Resonance, MSTS – Musculoskeletal Tumor Society, MTSS – Musculoskeletal Tumor Society Score, PCL – Polycaprolactone, PEG – Percutaneous Endoscopic Gastrostomy, PLA – Polylactic acid, PMMA – Polymethyl Methacrylate, PTX – Paclitaxel, PU – Polyurethane, PVA – Polyvinyl Alcohol, RIS – radioactive ^{125}I seed, RoM – Range of Motion, SEM – Scanning Electron Microscopy, SF-36 – 36-Item Short Form Survey, SLA – Stereolithography, SLS – Selective Laser Sintering, TESS – Toronto Extremity Salvage Score, TPU – Thermoplastic Polyurethane. **The authors are unfamiliar with the stated technolog*
